# Use of Telemedicine and Quality of Care Among Medicare Enrollees With Serious Mental Illness

**DOI:** 10.1001/jamahealthforum.2023.3648

**Published:** 2023-10-27

**Authors:** Andrew D. Wilcock, Haiden A. Huskamp, Alisa B. Busch, Sharon-Lise T. Normand, Lori Uscher-Pines, Pushpa V. Raja, Jose R. Zubizarreta, Michael L. Barnett, Ateev Mehrotra

**Affiliations:** 1 Department of Health Care Policy, Harvard Medical School, Boston, Massachusetts; 2 McLean Hospital, Belmont, Massachusetts; 3 Department of Biostatistics, Harvard T.H. Chan School of Public Health, Boston, Massachusetts; 4 RAND Health, Arlington, Virginia; 5 Department of Mental Health, Veterans Affairs Greater Los Angeles Healthcare System, Los Angeles, California; 6 Department of Statistics, Harvard University, Cambridge, Massachusetts; 7 Department of Health Policy and Management, Harvard T.H. Chan School of Public Health, Boston, Massachusetts; 8 Department of Medicine, Brigham and Women’s Hospital, Boston, Massachusetts; 9 Department of Medicine, Beth Israel Deaconess Medical Center, Boston, Massachusetts

## Abstract

**Importance:**

During the COVID-19 pandemic, a large fraction of mental health care was provided via telemedicine. The implications of this shift in care for use of mental health service and quality of care have not been characterized.

**Objective:**

To compare changes in care patterns and quality during the first year of the pandemic among Medicare beneficiaries with serious mental illness (schizophrenia or bipolar I disorder) cared for at practices with higher vs lower telemedicine use.

**Design, Setting, and Participants:**

In this cohort study, Medicare fee-for-service beneficiaries with schizophrenia or bipolar I disorder were attributed to specialty mental health practices that delivered the majority of their mental health care in 2019. Practices were categorized into 3 groups based on the proportion of telemental health visits provided during the first year of the pandemic (March 2020-February 2021): lowest use (0%-49%), middle use (50%-89%), or highest use (90%-100%). Across the 3 groups of practices, differential changes in patient outcomes were calculated from the year before the pandemic started to the year after. These changes were also compared with differential changes from a 2-year prepandemic period. Analyses were conducted in November 2022.

**Exposure:**

Practice-level use of telemedicine during the first year of the COVID-19 pandemic.

**Main Outcomes and Measures:**

The primary outcome was the total number of mental health visits (telemedicine plus in-person) per person. Secondary outcomes included the number of acute hospital and emergency department encounters, all-cause mortality, and quality outcomes, including adherence to antipsychotic and mood-stabilizing medications (as measured by the number of months of medication fills) and 7- and 30-day outpatient follow-up rates after discharge for a mental health hospitalization.

**Results:**

The pandemic cohort included 120 050 Medicare beneficiaries (mean [SD] age, 56.5 [14.5] years; 66 638 females [55.5%]) with serious mental illness. Compared with prepandemic changes and relative to patients receiving care at practices with the lowest telemedicine use: patients receiving care at practices in the middle and highest telemedicine use groups had 1.11 (95% CI, 0.45-1.76) and 1.94 (95% CI, 1.28-2.59) more mental health visits per patient per year (or 7.5% [95% CI, 3.0%-11.9%] and 13.0% [95% CI, 8.6%-17.4%] more mental health visits per year, respectively). Among patients of practices with middle and highest telemedicine use, changes in adherence to antipsychotic and mood-stabilizing medications were −0.4% (95% CI, −1.3% to 0.5%) and −0.1% (95% CI, −1.0% to 0.8%), and hospital and emergency department use for any reason changed by 2.4% (95% CI, −1.5% to 6.2%) and 2.8% (95% CI, −1.2% to 6.8%), respectively. There were no significant differential changes in postdischarge follow-up or mortality rates according to the level of telemedicine use.

**Conclusions and Relevance:**

In this cohort study of Medicare beneficiaries with serious mental illness, patients receiving care from practices that had a higher level of telemedicine use during the COVID-19 pandemic had more mental health visits per year compared with prepandemic levels, with no differential changes in other observed quality metrics over the same period.

## Key Points

**Question:**

Was telemedicine use during the COVID-19 pandemic associated with more visits and higher quality of care for patients with serious mental illness?

**Findings:**

In this cohort study of 120 050 Medicare beneficiaries with schizophrenia or bipolar I disorder, patients receiving mental health care at practices that almost exclusively switched to telemental health service had 13.0% more mental health visits than those receiving care at practices that largely used in-person visits. There were no changes in medication adherence, hospital and emergency department use, or mortality based on the extent of telemental health use.

**Meaning:**

These findings suggest that greater telemental health use was associated with more mental health visits, but not other changes in measures of quality of care.

## Teaser

This cohort study examines the use of telemental health services by Medicare beneficiaries with schizophrenia or bipolar I disorder, and whether outcomes differ according to a practice’s extent of telemedicine use.

## Introduction

The onset of the COVID-19 pandemic abruptly disrupted in-person care, and many mental health specialists switched to telemental health services (both audio-only and video visits).^1^ This shift was facilitated by expansions in reimbursement and many regulatory changes.^2^ The clinical impact of this switch to telemedicine is still unclear. Some patients and clinicians have expressed concern that the switch to telemedicine leads to lower quality care.^3,4^ On the other hand, it is also possible that the convenience of telemental health services could result in additional visits, which in turn could foster medication adherence and deter unnecessary emergency department (ED) visits and hospitalizations.^5^

Patients with serious mental illness (SMI), such as schizophrenia and bipolar I disorder, may be particularly suited for an assessment of the impact of telemental health. Among patients with SMI,^6,7^ continuity of care is particularly important because care disruptions are associated with worse patient outcomes.^7,8,9,10^ Furthermore, this population has high rates of ED visits and hospitalization, and medication adherence is a key component of care. This context makes it easier to assess whether telemental health services impact treatment adherence and deter acute crises. Patients with SMI tend to have lower income, are more likely to have unstable housing, and have fewer social supports, which may make telemedicine access more difficult^11,12,13^; however, prior work has highlighted that telemedicine for patients with SMI was quite common during the COVID-19 pandemic.^1,14^

Several randomized trials have shown that telemental health use results in outcomes comparable to those with in-person care,^15,16,17^ particularly for patients with depression.^18,19,20^ There is relatively less research on telemedicine for the treatment of SMI.^21,22,23^ In 1 trial, there were no differences in symptoms among 118 patients with SMI randomly assigned to an intervention of audio-only telemedicine visits every 2 weeks and individualized text messages every week.^24^ One observational study found that greater telemedicine use for patients with SMI was associated with modest increases in regular contact with mental health specialists.^25^ Prior research has been limited by small sample sizes, modest use of telemedicine compared with its level of adoption during the pandemic, or a focus only on residents in rural areas. To address these gaps in knowledge, we investigated the implications of greater telemedicine use by mental health specialty practices during the COVID-19 pandemic for changes in outpatient mental health visits and quality of care among Medicare beneficiaries with SMI.

## Methods

### Overview

Harvard Medical School’s Institutional Review Board approved this cohort study and waived the informed consent requirement because the use of deidentified administrative records presented little additional risk to privacy. We followed the Strengthening the Reporting of Observational Studies in Epidemiology (STROBE) reporting guideline.

We evaluated the care of Medicare beneficiaries with SMI (schizophrenia and bipolar I disorder) receiving care at specialty mental health practices that predominately used telemedicine during the COVID-19 pandemic compared with practices that relied more heavily on in-person services. We used the start of the pandemic in March 2020 as a natural experiment. For a variety of reasons including organizational resources, practice leadership, and clinician comfort with technology, some practices embraced telemedicine to a much greater degree than others.^3,26,27^

This study used a multicohort approach to compare changes in care experienced by a cohort of patients with SMI who received mental health care from the year prior to the pandemic through the first year of the pandemic (2019 to 2020; hereafter, the pandemic cohort). To understand whether these changes were different than what is typically observed year-over-year (for example, medication adherence typically decreases over time), we measured changes in care in a cohort of patients who received mental health care 2 years prior to the pandemic (2018 to 2019; hereafter, the prepandemic cohort), when there was little telemedicine use (eMethods 1 in Supplement 1).

### Pandemic and Prepandemic Cohorts

The pandemic and prepandemic cohorts of patients with SMI were followed up for 2 years. For the pandemic cohort, we assessed changes in outcomes from the year before the start of the COVID-19 pandemic (year 1, March 2019-February 2020) to the next year (year 2, March 2020-February 2021). For the prepandemic cohort, we assessed changes in outcomes from March 2018 to February 2019 (year 1) and from March 2019 to February 2020 (year 2). By design, patients were required to survive year 1; therefore, the mortality rate was captured only in year 2 for each cohort.

For the pandemic cohort, using Medicare Beneficiary Summary Files and Standard Analytic files, we identified beneficiaries with SMI who were continuously enrolled in traditional Medicare Part A and Part B from January 2019 through February 2021 (or through their death). Patients were included if they had at least 2 outpatient visits in 2019 with an *International Statistical Classification of Diseases and Related Health Problems, Tenth Revision *(*ICD-10*) code for schizophrenia and related disorders (*ICD-10* codes F20-F29) or bipolar I disorder (*ICD-10* codes F30, F31.0-F31.7) in any diagnosis field on outpatient claims (including outpatient facility claims and Medicare Part B carrier claims) or 1 inpatient claim with a first or second diagnosis for these conditions. Patients with both schizophrenia and bipolar I diagnoses were categorized as having schizophrenia. We used the same criteria to identify the prepandemic cohort in 2018.

Using Master Beneficiary Summary Files, we extracted demographic and prior disease burden information from each cohort’s baseline year (2019 for the pandemic cohort, 2018 for the prepandemic cohort) including age, sex, race and ethnicity, and the total number of the 27 Chronic Conditions Data Warehouse indicators^28^ for chronic conditions for each patient (eMethods 2 in Supplement 1). Medicare’s race and ethnicity information was obtained from the Social Security Administration, which captures self-reported race on applications for a social security number.^29^ Racial and ethnic categories included Asian or Pacific Islander, Black, Hispanic, White, and other (American Indian, Alaska Native, and unknown). Race and ethnicity were assessed given their historical association with differences in access to health services and health.

### Mental Health Specialty Practices

We focused on care provided by mental health specialty practices given that most patients with SMI are treated by mental health specialists. These specialists were defined using Centers for Medicare & Medicaid Services specialty codes for psychiatrists, psychologists, clinical psychologists, neuropsychiatrists, and licensed clinical social workers (codes 26, 62, 68, 86, and 80, respectively), as well as nurse practitioners who focus on mental health (psychiatric mental health nurse practitioners) as described by Richard et al.^30^ Visits to licensed professional counselors or marriage and family therapists are not reimbursed by Medicare and therefore were excluded. Consistent with prior work, practices were identified based on their unique federal tax identification numbers^31^; we identified outpatient mental health visits as those with a relevant *Current Procedural Terminology* (*CPT*) or Healthcare Common Procedure Coding System (HCPCS) code (eMethods 3 in Supplement 1) and a mental health diagnosis or substance use disorder (*ICD-10* codes F10.X to F60.X or F90.X, excluding F17.X nicotine dependence).^1^ Visits for services such as Assertive Community Training programs, peer support, or skills training and development, which are covered in some states by Medicaid for beneficiaries with dual eligibility, were excluded. Mental health specialty practices included in the study provided at least 1 mental health specialty visit during the study period. For each patient, all such service lines from a given mental health specialty practice on a given date were considered as a single mental health visit.

Patients from each cohort were attributed to mental health specialty practices based on which practice delivered most (>50%) of their specialty mental health visits during the baseline year (2019 for the pandemic cohort, 2018 for the prepandemic cohort). Patients not attributed to a practice and those with no mental health visits in 2019 were excluded from analyses.

We made several practice-level exclusions to ensure that practices had a substantial sample to characterize their care patterns and they were primarily outpatient mental health practices. We excluded practices that provided 30 or fewer mental health visits in any study year; provided no visits in any quarter (to ensure practices were open throughout the study period); did not treat patients with SMI during the pandemic and prepandemic periods; and provided any care in nursing homes, hospitals, or federally qualified or rural health centers in 2019. A total of 13 586 practices (49% of all practices identified) and 353 848 patients (69%) were excluded from the final sample (eFigure 1 in Supplement 1). The final set of practices was the same in both cohorts.

### Categorizing Practice Uptake of Telemedicine

Among the practices included, we measured the proportion of all their mental health visits that were provided via telemedicine in year 1 of the pandemic (March 2020-February 2021). Telemental health visits were defined as mental health visits during which at least 1 service was provided with a Medicare place of service code 02; HCPCS code G2025; HCPCS modifier codes GT, GQ, or 95; or *CPT* codes 99441-99443 and 98966-98968 (for audio-only services). Based on practices’ proportion of telemedicine use, we divided them into 3 groups (eFigure 2 in Supplement 1) with a similar number of practices: lowest telemedicine use (0%-49% telemental health visits), middle telemedicine use (50%-89% telemental health visits), and highest telemedicine use (90%-100% telemental health visits). Practices were assigned the same group for both the prepandemic and pandemic cohorts.

### Matching Practices

Practice characteristics of the 3 groups before matching are provided in eTable 1 in Supplement 1. Given baseline differences among the 3 groups of practices, we used matching to balance observable practice characteristics. We used profile matching^32^ to find the largest sample of practices in each group whereby characteristics were balanced compared with the overall practice characteristics in the full sample. This method optimizes the size of the matched sample and directly balances covariates across all 3 groups. Matching was based on 2019 Medicare Part B carrier data on practice location (Northeast, Midwest, South, West), practice size (unique clinicians based on National Provider Identifier billing at practice and categorized as 1, 2-9, or ≥10 clinicians), mental health focus (proportion of visits for patients with a mental health diagnosis), urbanicity (proportion of visits delivered from a metropolitan area), and practice Medicaid share (proportion of visits for beneficiaries with dual eligibility for Medicaid).

### Outcomes

The primary outcome was the total number of mental health visits per person. For each patient, we measured the following factors over each study year to assess beneficiaries’ health care use overall: number of mental health visits, mental health visits with a specialist, and acute care encounters (hospitalization and ED visits) for any reason and for mental illness specifically. These outcomes measured beneficiary utilization overall, not just services delivered by their attributed practices. We did not separately examine video and audio-only visits given concerns about the ability to distinguish between these visits in claims.^33^ Secondary quality outcomes were assessed over each study year and included medication adherence (months with a filled prescription for antipsychotic or mood-stabilizing medication); receipt of a minimum number of specialty mental health visits (≥1 visit every 6 months, ≥1 visit every 3 months); and whether patients received a follow-up mental health visit within 7 or 30 days of discharge for a mental health hospitalization. We also captured all-cause mortality in year 2 and noted whether patients switched to a different specialty mental health practice in year 2. For the medication adherence measure only, we required that patients have continuous Medicare Part D coverage (85% of the full sample). Details on measurement of these outcomes and justification for the choice of health services and quality measures are included in eMethods 2 in Supplement 1.

### Statistical Analysis

Data were analyzed at the patient level. For each cohort, we measured changes from year 1 to year 2. A linear regression model was used to assess the change in each primary and secondary outcome based on categorical variables for treatment (ie, the 3 groups according to lowest, middle, and highest telemental health use), the cohort, an interaction between treatment group and cohort, and year 1 demographic characteristics. The SEs were clustered at the practice level. Full specifications of our model are provided in eMethods 4 in Supplement 1. Logistic regression was used to investigate mortality, which had a low probability of occurring and for which a linear model may not have been appropriate.

In addition to these statistical models, we described unadjusted trends in mental health visits, acute care use, and medication fills at the patient level according to a practice’s telemedicine group (lowest, middle, or highest telemedicine use). Mean outcomes over years 1 and 2 for the pandemic cohort were overlaid on mean outcomes over years 1 and 2 for the prepandemic cohort to illustrate differences in trends before and after the beginning of the pandemic. We assessed the absolute and relative differences in trends in the 3 telemedicine use groups to ascertain whether changes from usual care patterns were associated with greater telemedicine use by practices.

There have been long-standing concerns that some patient groups, such as those in rural areas or those with lower incomes, have particularly poor access to mental health care.^34,35^ To understand whether differential changes in visits were seen among these underserved groups, we conducted exploratory analyses to assess for heterogeneity in changes in visits by schizophrenia vs bipolar I disorder diagnosis, age group (<40, 40-54, 55-64, 65-74, and ≥75 years), race and ethnicity, sex, urbanicity, and disease burden (0-1, 2-6, 7-9, or ≥10 comorbidities). Because the sample size for many racial and ethnic groups was insufficient, we compared White patients with all other patients. Details are provided in eMethods 4 in Supplement 1.

We conducted all analyses in Stata, version 17.0 (StataCorp LLC) and R, version 3.5.0 (R Foundation for Statistical Computing). Model estimates are reported with 95% CIs. Analyses were conducted in November 2022.

## Results

The matched analytic sample included a total of 247 707 Medicare beneficiaries with SMI (120 050 patients in the pandemic cohort and 127 657 patients in the prepandemic cohort). Of the patients in the pandemic cohort (mean [SD] age, 56.6 [14.5] years; 66 638 females [55.5%] and 53 412 males [44.5%]), 1848 were Asian or Pacific Islander individuals (1.5%), 16 284 were Black individuals (13.6%), 3633 were Hispanic individuals (3.0%), 90 983 were White individuals (78.2%), and 4347 were individuals of other race or ethnicity (3.6%). Of the 11 170 practices included in the sample, 3180 had the lowest level of telemedicine use (0%-49% telemental health visits), 3793 had middle telemedicine use (50%-89% telemental health visits), and 4197 had highest telemedicine use (90%-100% telemental health visits) (Table 1). From March 2020 to February 2021, the mean (SD) proportion of mental health visits provided via telemedicine was 14.6% (16.7%) in practices with the lowest use, 74.0% (18.8%) in those with middle use, and 94.6% (2.3%) in those with highest use. Across the 3 groups of matched practices, practice and patient characteristics were balanced (ie, <0.1 standardized mean difference) (Table 1).

**Table 1.** Mental Health Specialty Practice and Patient Characteristics in the Pandemic Cohort After Matching

Characteristic	No. (%)				Absolute SMD by level of telemedicine use^b^	
	Matched sample overall	Lowest telemedicine use^a^	Middle telemedicine use^a^	Highest telemedicine use^a^	Lowest vs middle	Lowest vs highest
2019 Practices						
Total practices	11 170 (100)	3180 (28.5)	3793 (33.9)	4197 (37.6)	NA	NA
Mental health visits per practice, mean (SD)	520 (994)	475 (867)	553 (1075)	523 (1007)	0.080	0.052
Telemedicine share in year 1 of pandemic, mean % (SD)	64.8 (34.8)	14.6 (16.7)	74.0 (11.8)	94.6 (2.3)	NA	NA
Practice location						
Northeast	3586 (32.1)	962 (30.3)	1161 (30.6)	1460 (34.8)	0.008	0.097
Midwest	2189 (19.6)	639 (20.1)	792 (20.9)	763 (18.2)	0.019	0.049
South	3329 (29.8)	1013 (31.9)	1168 (30.8)	1149 (27.4)	0.023	0.098
West	2066 (18.5)	566 (17.8)	672 (17.7)	825 (19.7)	0.002	0.048
No. of clinicians in the practice						
1	8439 (75.6)	2449 (77.0)	2760 (72.8)	3230 (77.0)	0.098	0.001
2-9	2138 (19.1)	598 (18.8)	789 (20.8)	751 (17.9)	0.050	0.024
≥10	593 (5.3)	133 (4.2)	244 (6.4)	216 (5.1)	0.100	0.046
Share of visits with a mental health focus, mean % (SD)	95.9 (12.1)	95.4 (14.1)	95.7 (12.3)	96.6 (10.1)	0.017	0.061
Share of visits delivered from urban location, mean % (SD)	88.5 (31.8)	87.0 (33.6)	87.9 (32.4)	90.2 (29.6)	0.028	0.100
Share of visits delivered to patients with Medicaid, mean % (SD)	30.2 (27.5)	31.3 (28.6)	30.9 (27.1)	28.6 (26.9)	0.013	0.098
2019 Patient characteristics						
Patients with SMI	120 050 (100)	30 025 (25.0)	48 126 (40.1)	41 899 (34.9)	NA	NA
With schizophrenia	59 785 (49.8)	15 211 (50.7)	24 270 (50.4)	20 354 (48.6)	0.005	0.042
With bipolar I disorder	60 265 (50.2)	14 814 (49.3)	23 856 (49.6)	21 545 (51.4)	0.005	0.042
Age, mean (SD), y	56.5 (14.5)	57.2 (14.6)	55.7 (14.4)	56.8 (14.5)	0.096	0.022
Age group, y						
<40	18 873 (15.7)	4446 (14.8)	8011 (16.6)	6416 (15.3)	0.051	0.014
40-54	33 008(27.5)	7997 (26.6)	13 842 (28.8)	11 169 (26.7)	0.048	0.001
55-64	29 726 (24.8)	7375 (24.6)	11 988 (24.9)	10 363 (24.7)	0.008	0.004
65-74	27 924 (23.3)	7275 (24.2)	10 426 (21.7)	10 223 (24.4)	0.061	0.004
≥75	10 519 (8.8)	2932 (9.8)	3859 (8.0)	3728 (8.9)	0.061	0.030
Race and ethnicity						
Asian or Pacific Islander	1848 (1.5)	535 (1.8)	688 (1.4)	625 (1.5)	0.028	0.023
Black	16 284 (13.6)	4197 (14.0)	6852 (14.2)	5235 (12.5)	0.007	0.044
Hispanic	3633 (3.0)	874 (2.9)	1278 (2.7)	1481 (3.5)	0.016	0.035
White	93 938 (78.2)	23 411(78.0)	37 514 (77.9)	33 013 (78.8)	0.001	0.020
Other^c^	4347 (3.6)	1008 (3.4)	1794 (3.7)	1545 (3.7)	0.020	0.018
Sex						
Male	53 412 (44.5)	13 543 (45.1)	21 550 (44.8)	18 319 (43.7)	0.007	0.028
Female	66 638 (55.5)	16 482 (54.9)	26 576 (55.2)	23 580 (56.3)	0.007	0.028
Rural residence	22 706 (18.9)	5450 (18.2)	10 336 (21.5)	6920 (16.5)	0.084	0.043
Dually enrolled in Medicaid	75 794 (63.1)	18 969 (63.2)	31 120 (64.7)	25 705 (61.3)	0.031	0.038
Original Medicare eligibility based on disability	99 072 (82.5)	24 495 (81.6)	40 449 (84.0)	34 128 (81.5)	0.065	0.003
Enrolled in Medicare Part D	99 459 (82.8)	24 718 (82.3)	39 975 (83.1)	34 766 (83.0)	0.020	0.017
Chronic conditions						
0-1	14 290 (11.9)	3274 (10.9)	6067 (12.6)	4949 (11.8)	0.053	0.029
2-6	52 475 (43.7)	12 684 (42.2)	21 510 (44.7)	18 281 (43.6)	0.049	0.028
7-9	27 401 (22.8)	6927 (23.1)	10 724 (22.3)	9750 (23.3)	0.019	0.005
≥10	25 884 (21.6)	7140 (23.8)	9825 (20.4)	8919 (21.3)	0.081	0.060

Abbreviations: NA, not applicable; SMD, standardized mean difference; SMI, serious mental illness.

^a^ Practices were categorized based on the proportion of telemental health visits relative to all mental health visits (telemedicine and in-person visits) provided during the first year of the pandemic (March 2020-February 2021) into 3 groups: lowest telemedicine use (0%-49% TMH visits), middle telemedicine use (50%-89% TMH visits), or highest telemedicine use (90%-100% TMH visits).

^b^ SMDs greater than 0.1 indicate a sizeable difference.

^c^ Other race includes American Indian, Alaska Native, and unknown.

### Trends in Outcomes

**Figure 1.**
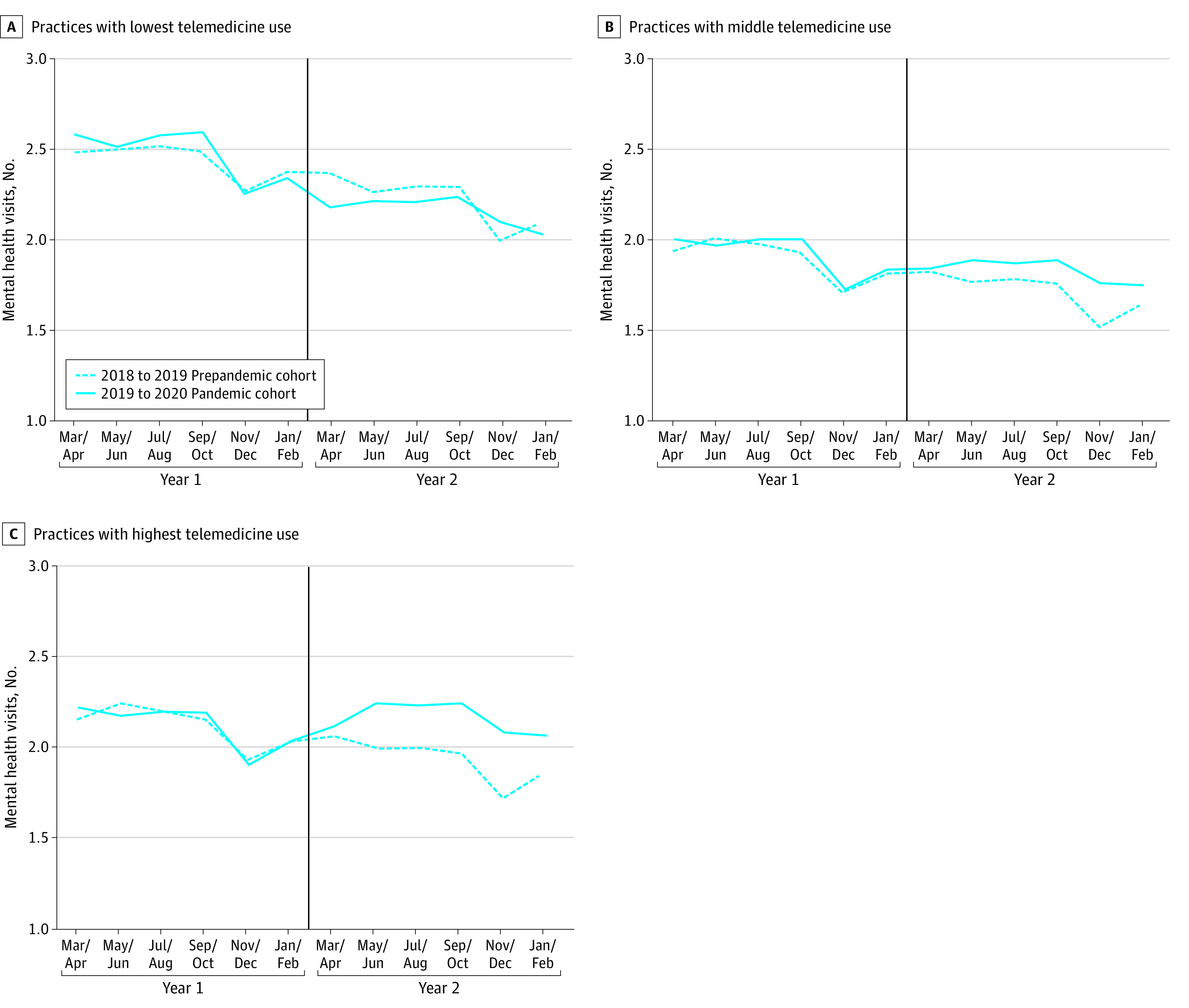
Two-Year Trends in the Mean Number of Mental Health Visits per Patient According to Practices’ Use of Telemedicine Two-year trends in unadjusted mean number of mental health visits (including video and audio-only services) for patients in the prepandemic and pandemic cohorts. Practices were categorized based on the proportion of telemental health visits provided during the first 12 months of the COVID-19 pandemic; those with the lowest telemedicine use had 0% to 49% telemental health visits, those with middle telemedicine use had 50%-89% telemental health visits, and those with highest telemedicine use had 90%-100% telemental health visits. Vertical lines separate years 1 and 2 and correspond to the start of the pandemic in March 2020 for the pandemic cohort.

**Figure 2.**
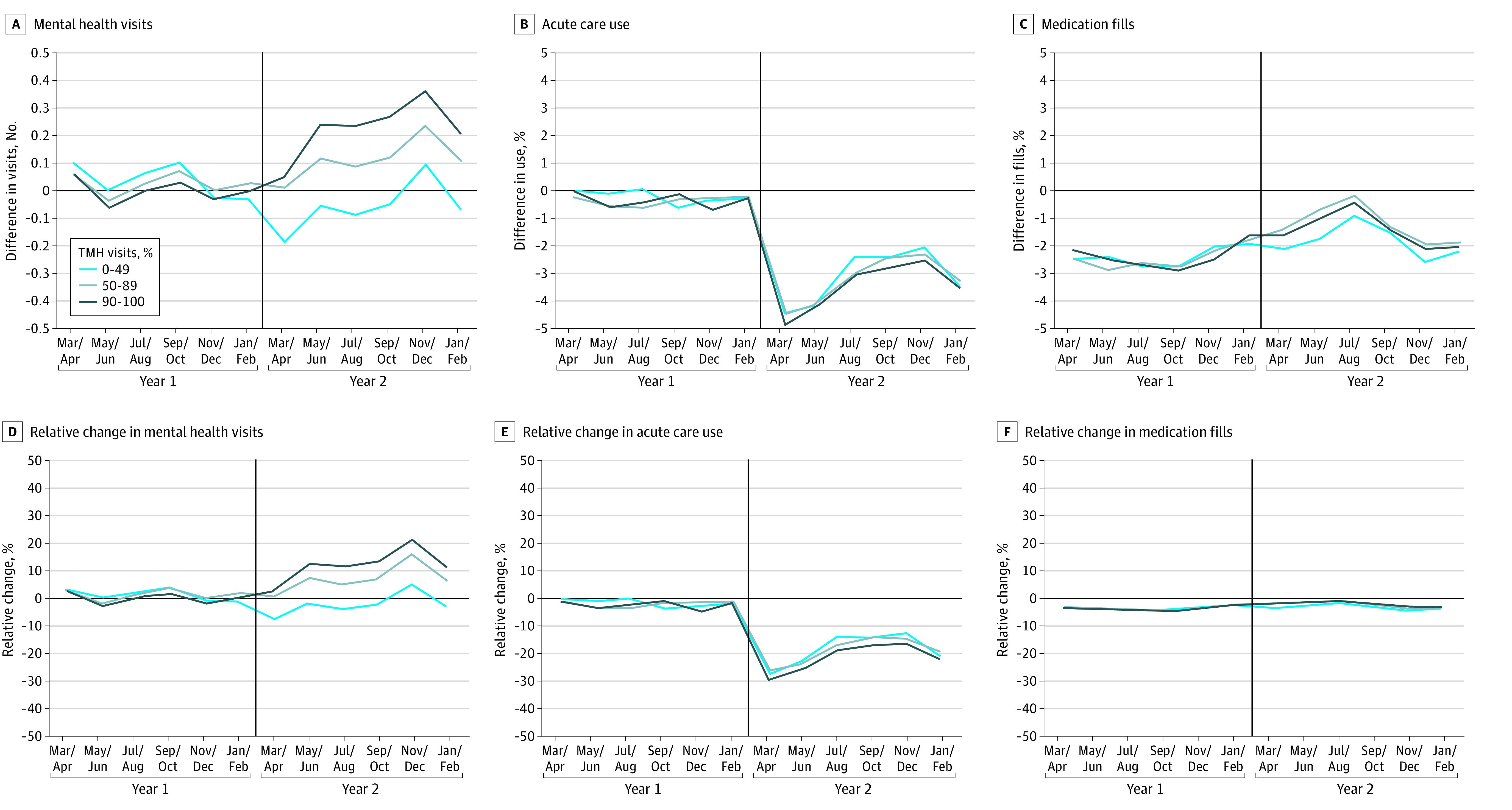
Differences in 2-Year Trends Between Pandemic and Prepandemic Cohorts in Mental Health Visits, Acute Care Use, and Medication Adherence According to Practices’ Use of Telemedicine Plots show the differences in the unadjusted mean trends in mental health visits, acute care use (hospitalization and emergency department visits), and antipsychotic and mood-stabilizing medication fills between the pandemic and prepandemic cohorts. Panels A-C show absolute differences in trends between the pandemic and prepandemic cohorts (difference = pandemic − prepandemic values) according to the practice’s use of telemedicine. Practices with lowest, middle, and highest use delivered 0%-49%, 50%-89%, and 90%-100% of mental health visits, respectively, via telemedicine. Panels D-F show the differences as relative changes compared with the prepandemic cohort mean (relative change = difference/prepandemic value). Vertical lines separate years 1 and 2 and correspond to the start of the pandemic in March 2020 for the pandemic cohort. TMH indicates telemental health.

Trends in mental health visits in year 1 were not different between the pandemic and prepandemic cohorts (Figure 1). Among patients in the pandemic cohort, after the COVID-19 public health emergency was declared in March 2020, the mean proportion of telemental health visits was higher in the middle telemedicine and highest telemedicine use groups than in the prepandemic cohort (Figure 1). No clear patterns of relative unadjusted differences between the pandemic and prepandemic cohorts were seen for acute care encounters or medication adherence across telemedicine use groups (Figure 2).

### Estimated Changes in Outcomes by Practice Uptake of Telemedicine

We observed no differential changes in outcomes in year 1 based on practice uptake of telemedicine (eMethods 5 and eTable 2 in Supplement 1). Similarly, we did not find any differential changes in patient characteristics in year 2 (eTable 3 in Supplement 1).

Relative to the differences in year 1 to year 2 changes in the number of mental health visits observed among practices with the lowest telemedicine use between the prepandemic and pandemic cohorts, patients who received care at middle telemedicine use practices had a differential absolute increase of 1.11 (95% CI, 0.45-1.76) visits per year, and patients who received care at highest telemedicine use practices had a differential absolute increase of 1.94 (95% CI, 1.28-2.59) visits per year (Table 2). These increases represent a 7.5% (95% CI, 3.0%-11.9%) increase and a 13.0% (95% CI, 8.6%-17.4%) increase relative to the number of visits for patients attributed to practices with the lowest telemedicine use in year 1 for the pandemic cohort (14.85 visits). The increase in specialty mental health visits was larger for counseling and therapy visits than for mental health prescriber visits (eResults in Supplement 1).

**Table 2.** Patient-Level Adjusted Differences in Mental Health Services and Changes in Outcomes by Practice Level of Telemedicine Use

Outcome	Practices with lowest telemedicine use^a^		Practices with middle telemedicine use^a^		Practices with highest telemedicine use^a^		Differential in changes by telemedicine use, No. (95% CI)	
	Year 1, mean (SD)	Differential (95% CI)	Year 1, mean (SD)	Differential (95% CI)	Year 1, mean (SD)	Differential (95% CI)	Middle vs lowest	Highest vs lowest
Total No. of mental health visits	14.85 (22.53)	−0.60 (−1.20 to 0.01)	11.52 (14.85)	0.51 (0.25 to 0.77)	12.72 (16.28)	1.34 (1.07 to 1.60)	1.11 (0.45 to 1.76)	1.94 (1.28 to 2.59)
Visits delivered by mental health specialists	13.97 (22.24)	−0.60 (−1.27 to 0.07)	10.68 (14.50)	0.34 (0.10 to 0.59)	11.96 (15.97)	1.27 (1.01 to 1.53)	0.94 (0.23 to 1.66)	1.87 (1.15 to 2.59)
Acute care encounters^b^	1.61 (3.58)	−0.33 (−0.39 to −0.28)	1.57 (3.43)	−0.30 (−0.33 to −0.26)	1.45 (3.15)	−0.29 (−0.33 to −0.25)	0.04 (−0.02 to 0.10)	0.05 (−0.02 to 0.11)
Acute care encounters for mental illness	0.30 (1.23)	−0.06 (−0.08 to −0.04)	0.29 (1.10)	−0.04 (−0.05 to −0.02)	0.26 (1.05)	−0.03 (−0.04 to −0.01)	0.02 (0.0003 to 0.05)	0.03 (0.01 to 0.06)
Months with antipsychotic or mood-stabilizing medication fills	8.07 (4.51)	0.01 (−0.05 to 0.07)	8.13 (4.42)	−0.03 (−0.07 to 0.02)	8.11 (4.45)	0.01 (−0.04 to 0.05)	−0.04 (−0.11 to 0.04)	−0.01 (−0.08 to 0.07)
Minimum threshold of mental health visits, mean % (SD)								
≥1 Visit every 3 mo	59.9 (49.0)	−3.63 (−5.69 to −1.58)	59.9 (49.0)	−2.32 (−3.97 to −0.67)	63.1 (48.2)	−1.41 (−2.77 to −0.05)	1.31 (−1.32 to 3.94)	2.22 (−0.24 to 4.68)
≥1 Visit every 6 mo	87.4 (33.2)	−5.53 (−7.67 to −3.40)	87.8 (32.8)	−2.28 (−4.21 to −0.34)	89.2 (31.0)	−2.40 (−3.38 to −1.41)	3.26 (0.38 to 6.13)	3.14 (0.79 to 5.48)
No mental health visits in year 2, mean % (SD)^c^	12.9 (33.5)	4.34 (2.78 to 5.90)	13.0 (33.6)	3.07 (2.25 to 3.90)	11.5 (31.9)	2.49 (1.49 to 3.49)	−1.27 (−3.03 to 0.49)	−1.85 (−3.70 to −0.003)
Patient switched to new practice in year 2, mean % (SD)^c^	15.2 (35.9)	−0.13 (−1.08 to 0.82)	14.2 (34.9)	−0.80 (−1.57 to −0.04)	12.7 (33.3)	−1.43 (−1.98 to −0.89)	−0.67 (−1.89 to 0.55)	−1.30 (−2.39 to −0.21)
Follow-up after discharge, mean % (SD)								
Within 7 d	42.1 (49.4)	0.73 (−3.27 to 4.73)	40.3 (49.1)	1.09 (−1.76 to 3.94)	42.9 (49.5)	0.66 (−2.36 to 3.68)	0.36 (−4.55 to 5.27)	−0.07 (−5.08 to 4.94)
Within 30 d	69.8 (45.9)	1.12 (−3.25 to 5.48)	71.9 (45.0)	−0.49 (−3.28 to 2.30)	72.8 (44.5)	0.39 (−2.71 to 3.49)	−1.61 (−6.79 to 3.57)	−0.73 (−6.09 to 4.64)
Mortality rate, mean % (SD)^c^^,^^d^	2.9 (16.7)	0.97 (0.66 to 1.28)	2.8 (16.4)	0.62 (0.38 to 0.85)	2.6 (15.9)	0.57 (0.35 to 0.79)	−0.35 (−0.73 to 0.03)	−0.34 (−0.73 to 0.04)

Abbreviation: TMH, telemental health.

^a^ Practices were categorized based on the proportion of TMH visits relative to all mental health visits (TMH and in-person visits) provided during the first year of the pandemic (March 2020-February 2021) into 3 groups: lowest telemedicine use (0%-49% TMH visits), middle telemedicine use (50%-89% TMH visits), or highest telemedicine use (90%-100% TMH visits).

^b^ Hospitalization or emergency department visits.

^c^ These outcomes were only available in year 2 and represent year 2 differential changes between the pandemic and prepandemic cohorts. For these outcomes only, the values in the year 1 mean columns are year 2 means (SDs) from the prepandemic cohort.

^d^ Due to its low probability, mortality was modeled using logistic regression. All other outcomes used linear regression.

There was no differential change in acute care encounters for any reason among patients of middle telemedicine use (2.4%; 95% CI, −1.5% to 6.2%) and highest telemedicine use practices (2.8%; 95% CI, −1.2% to 6.8%) compared with patients of lowest telemedicine use practices. However, acute care encounters for mental illness were differentially higher among patients of middle telemedicine use (8.2%; 95% CI, 0.1%-16.4%) and highest telemedicine use practices (11.3%; 95% CI, 3.0%-19.5%). There was no differential change in medication adherence among the 3 practice groups (middle vs lowest telemedicine use practices: −0.4% [95% CI, −1.3% to 0.5%]; highest vs lowest telemedicine use practices: −0.1% [95% CI, −1.0% to 0.8%]).

**Figure 3.**
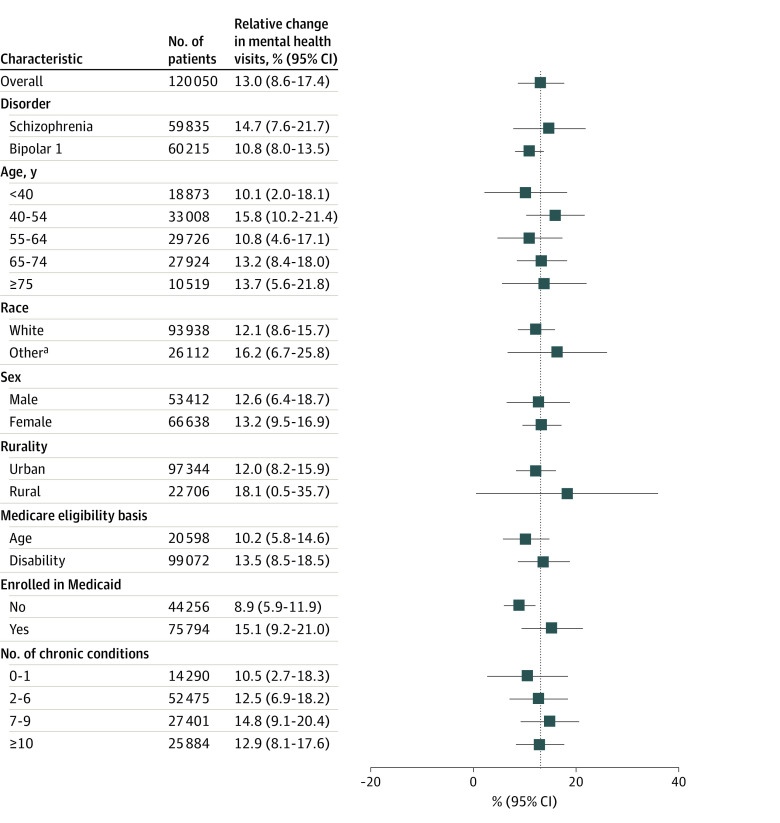
Relative Change in Mental Health Visits for Practices With Highest vs Lowest Use of Telemental Health by Patient Demographic Characteristics Boxes represent means and bars represent 95% CI. Vertical dashed line indicates overall change across the entire study population. ^a^Other race includes Asian or Pacific Islander, Black, Hispanic, American Indian, Alaska Native, and unknown race and ethnicity. Because the sample size for these racial and ethnic groups was insufficient for comparisons, we grouped them into this other category.

We did not observe differential rate changes in follow-up after discharge for a mental health hospitalization or all-cause mortality among the 3 telemedicine use groups. Compared with patients receiving care at lowest telemedicine use practices, patients receiving care at highest telemedicine use practices more often had at least 1 mental health visit every 6 months (3.6%; 95% CI, 0.9% to 6.3%) and less often had no visits in year 2 (−14.4%; 95% CI, −28.8% to −0.02%). Among patients with at least 1 visit in year 2, again compared with patients receiving care at lowest telemedicine use practices, patients of highest telemedicine use practices less often switched to a new specialty mental health practice (−8.6%; 95% CI, −15.7% to −1.4%). Model results were nearly identical to alternative specifications that included year 1 outcomes as an explanatory variable. The relative increase in mental health visits between the highest and lowest telemedicine use practices did not substantially differ by demographic characteristics or disease burden (Figure 3).

## Discussion

During the COVID-19 pandemic, patients receiving care at mental health practices that switched almost exclusively to telemedicine had more outpatient mental health visits than patients at practices that continued to rely largely on in-person visits. Patients receiving care at practices with the highest telemedicine use more often received at least a minimum number of visits and less often switched practices. We did not find differential changes across other utilization or quality outcomes including months with medication fills, outpatient follow-up after mental health hospitalization, or all-cause mortality. Together, our results suggest that, during a period when practices rapidly adopted telemedicine, patients in practices with a high use of telemedicine had more visits with their clinicians and did not appear to experience some of the adverse outcomes that might be expected if telemedicine visits were a poor substitute for in-person care.

This study contributes to the growing literature on the implications of adopting telemedicine for the treatment of mental illness. Our findings are consistent with prior randomized trials for depression and other mental illnesses in which patients randomly assigned to receive telemental health vs in-person visits had similar outcomes.^21,22,23^ Our results are also consistent with prior observational studies that found that patients had more visits if they lived in rural counties with more telemedicine practices or were cared for by health centers with greater telemedicine use.^25,36^ Given the use of a national sample, including both rural and urban patients, and a larger set of quality health care use measures, we believe our study offers the largest, most generalizable evidence yet on the clinical implications of telemedicine for the care of mental illness.

One surprising finding was that patients receiving care from practices with the highest telemedicine use had more hospitalizations and ED visits for mental health conditions than patients of practices with the lowest telemedicine use. This finding could be interpreted as a sign that greater telemedicine use was associated with a lower quality of care. An alternative explanation may be that the underlying assumption that, at a population level, more ED visits and hospitalizations are a sign of poor quality of care is incorrect.^5^ In some circumstances, increased hospital use might be a sign of better care in that patients in acute crisis were correctly identified and stabilized. This finding is consistent with several prior studies that also found that telemedicine use was associated with more ED visits or hospitalizations.^25,37,38,39^ We also recognize that hospitalizations and ED visits for all indications fell sharply early in the pandemic. In that context, one must be cautious in interpreting relative changes in hospitalizations and ED use.

Our findings have several implications for clinical practice and policy. While clinical experience and the literature suggest potential barriers at the patient level that could prevent individuals with SMI from effectively accessing care via telemedicine, our results highlight that many practices were successfully able to engage with these patients during the pandemic. The finding that practices that almost exclusively used telemedicine had outcomes similar to practices relying on in-person visits may reassure patients and clinicians who have been concerned that telemedicine may result in inferior quality as well as buttress recent policy decisions to permanently expand payment for telemental health services in the Medicare program and in several states.^40,41^

We interpreted the increase in mental health visits as a favorable change given known access barriers among patients with SMI and the importance of continuity of care in this population. The important caveat is that the increase in mental health visits that we observed was not associated with increased medication adherence or fewer hospitalizations or ED visits. Furthermore, we did not observe greater increases in mental health visits among certain patient subgroups, such as patients with low income or those living in rural areas, who often face the greatest barriers in accessing care. It is important to emphasize this is a single study and more research is needed to understand the implications of telemedicine for other clinical areas as well as for other outcomes that we could not capture in our data.

### Limitations

This study has several key limitations. First, our findings may not generalize to all patients with SMI or to those with other mental illnesses. We focused on patients in the Medicare fee-for-service program with established care relationships, and we did not include practices that work in facilities such as nursing homes or federally qualified health centers. Therefore, our findings may not generalize to uninsured patients with SMI or to those covered under commercial or Medicaid-only health plans. In addition, our cohorts included patients with dual eligibility for Medicare and Medicaid; therefore, we could not assess the association between telemedicine and mental health–related services and clinicians not covered by Medicare, such as Assertive Community Training program services or visits provided by family therapists. Second, the development of the patient cohort, patient characteristics, and outcomes were limited by the information captured in claims data, which are limited in the clinical and sociodemographic details they can provide. For example, we could not capture patient- or clinician-reported measures of clinical outcomes, such as symptom control. Furthermore, we did not have a sufficient sample size to explore differential changes across different racial or ethnic groups. Moreover, prior work^42^ has highlighted that there is poor accuracy of self-reported race and ethnicity data in the Medicare enrollment files, which limits their use in disparity comparisons. Finally, the analyses of heterogeneity in the changes across various subgroups did not account for multiple testing, and these findings should be considered exploratory.

## Conclusions

In this cohort study of Medicare beneficiaries with SMI, compared with practices that continued to provide mostly in-person care, specialty mental health practices that relied almost exclusively on telemedicine during the pandemic had more patient visits and outcomes were equivalent in terms of medication adherence, total acute care encounters, and mortality. Using telemedicine to care for patients with SMI may be an effective policy for provider organizations or health plans to improve patient-clinician engagement and continuity of care, which may help these patients better manage their conditions. However, given the current evidence, policy makers should not expect to realize concurrent cost savings from adopting telemedicine for this population.

## Notes

Supplement 1. **eMethods 1.** Details on Study Design **eMethods 2.** Patient Characteristics and Outcome Definitions **eMethods 3.** Details on Codes Used to Identify Visits **eMethods 4.** Details on Model Specifications **eMethods 5.** Year 1 Trends and Sample Composition Shifts **eResults.** Details on Specialty Mental Health Visits **eFigure 1.** Flowchart of Cohort Exclusions and Sample Sizes **eFigure 2.** Share of Total Mental Health Visits Delivered With Telemedicine Over the First Year of the Pandemic **eTable 1.** Characteristics of Specialty Mental Health Practices Before Matching **eTable 2.** Year 1 Trends in Outcomes **eTable 3.** Changes in Patient Characteristics from Year 1 to Year 2

Supplement 2.
Data Sharing Statement

## References

[1] Busch AB, Huskamp HA, Raja P, Rose S, Mehrotra A. Disruptions in care for Medicare beneficiaries with severe mental illness during the COVID-19 pandemic. JAMA Netw Open. 2022;5(1):e2145677. doi:10.1001/jamanetworkopen.2021.45677 35089352 PMC8800078

[2] Mehrotra A, Bhatia RS, Snoswell CL. Paying for telemedicine after the pandemic. JAMA. 2021;325(5):431-432. doi:10.1001/jama.2020.25706 33528545 PMC9320940

[3] Uscher-Pines L, Raja P, Qureshi N, Huskamp HA, Busch AB, Mehrotra A. Use of tele-mental health in conjunction with in-person care: a qualitative exploration of implementation models. Psychiatr Serv. 2020;71(5):419-426. doi:10.1176/appi.ps.201900386 31996115 PMC7271813

[4] SteelFisher GK, McMurtry CL, Caporello H, . Video telemedicine experiences in COVID-19 were positive, but physicians and patients prefer in-person care for the future. Health Aff (Millwood). 2023;42(4):575-584. doi:10.1377/hlthaff.2022.01027 37011316 PMC11154740

[5] Blanco C, Wall MM, Olfson M. Implications of telepsychiatry for cost, quality, and equity of mental health care. JAMA Psychiatry. 2022;79(12):1147-1148. doi:10.1001/jamapsychiatry.2022.3330 36260304

[6] Copeland LA, Zeber JE, Rosenheck RA, Miller AL. Unforeseen inpatient mortality among veterans with schizophrenia. Med Care. 2006;44(2):110-116. doi:10.1097/01.mlr.0000196973.99080.fb 16434909

[7] Davis CL, Kilbourne AM, Blow FC, . Reduced mortality among Department of Veterans Affairs patients with schizophrenia or bipolar disorder lost to follow-up and engaged in active outreach to return for care. Am J Public Health. 2012;102(suppl 1):S74-S79. doi:10.2105/AJPH.2011.300502 22390607 PMC3496434

[8] Bowersox NW, Kilbourne AM, Abraham KM, . Cause-specific mortality among veterans with serious mental illness lost to follow-up. Gen Hosp Psychiatry. 2012;34(6):651-653. doi:10.1016/j.genhosppsych.2012.05.014 22795048

[9] McCarthy JF, Blow FC, Valenstein M, . Veterans Affairs Health System and mental health treatment retention among patients with serious mental illness: evaluating accessibility and availability barriers. Health Serv Res. 2007;42(3 Pt 1):1042-1060. doi:10.1111/j.1475-6773.2006.00642.x 17489903 PMC1955257

[10] Fischer EP, McCarthy JF, Ignacio RV, . Longitudinal patterns of health system retention among veterans with schizophrenia or bipolar disorder. Community Ment Health J. 2008;44(5):321-330. doi:10.1007/s10597-008-9133-z 18401711

[11] Kozloff N, Mulsant BH, Stergiopoulos V, Voineskos AN. The COVID-19 global pandemic: implications for people with schizophrenia and related disorders. Schizophr Bull. 2020;46(4):752-757. doi:10.1093/schbul/sbaa051 32343342 PMC7197583

[12] Ayano G, Tesfaw G, Shumet S. The prevalence of schizophrenia and other psychotic disorders among homeless people: a systematic review and meta-analysis. BMC Psychiatry. 2019;19(1):370. doi:10.1186/s12888-019-2361-7 31775786 PMC6880407

[13] Jester DJ, Thomas ML, Sturm ET, ; Clinical Outcomes. Review of major social determinants of health in schizophrenia-spectrum psychotic disorders: I. Clinical outcomes. Schizophr Bull. 2023;49(4):837-850. doi:10.1093/schbul/sbad023 37022779 PMC10318890

[14] Raja PV, Gabrielian S, Doran N. Access to care for veterans with serious mental illness during the COVID-19 pandemic. Psychiatr Serv. 2021;72(11):1324-1327. doi:10.1176/appi.ps.202000898 34030456

[15] García-Lizana F, Muñoz-Mayorga I. What about telepsychiatry? a systematic review. Prim Care Companion J Clin Psychiatry. 2010;12(2):PCC.09m00831. 20694116 10.4088/PCC.09m00831whi PMC2911004

[16] Chakrabarti S. Usefulness of telepsychiatry: a critical evaluation of videoconferencing-based approaches. World J Psychiatry. 2015;5(3):286-304. doi:10.5498/wjp.v5.i3.286 26425443 PMC4582305

[17] Hilty DM, Ferrer DC, Parish MB, Johnston B, Callahan EJ, Yellowlees PM. The effectiveness of telemental health: a 2013 review. Telemed J E Health. 2013;19(6):444-454. doi:10.1089/tmj.2013.0075 23697504 PMC3662387

[18] Ruskin PE, Silver-Aylaian M, Kling MA, . Treatment outcomes in depression: comparison of remote treatment through telepsychiatry to in-person treatment. Am J Psychiatry. 2004;161(8):1471-1476. doi:10.1176/appi.ajp.161.8.1471 15285975

[19] Fortney JC, Pyne JM, Mouden SB, . Practice-based versus telemedicine-based collaborative care for depression in rural federally qualified health centers: a pragmatic randomized comparative effectiveness trial. Am J Psychiatry. 2013;170(4):414-425. doi:10.1176/appi.ajp.2012.12050696 23429924 PMC3816374

[20] García-Lizana F, Muñoz-Mayorga I. Telemedicine for depression: a systematic review. Perspect Psychiatr Care. 2010;46(2):119-126. doi:10.1111/j.1744-6163.2010.00247.x 20377799

[21] Zarate CA Jr, Weinstock L, Cukor P, . Applicability of telemedicine for assessing patients with schizophrenia: acceptance and reliability. J Clin Psychiatry. 1997;58(1):22-25. doi:10.4088/JCP.v58n0104 9055833

[22] Kasckow J, Felmet K, Appelt C, Thompson R, Rotondi A, Haas G. Telepsychiatry in the assessment and treatment of schizophrenia. Clin Schizophr Relat Psychoses. 2014;8(1):21-27A. doi:10.3371/CSRP.KAFE.021513 23428781 PMC4132656

[23] Santesteban-Echarri O, Piskulic D, Nyman RK, Addington J. Telehealth interventions for schizophrenia-spectrum disorders and clinical high-risk for psychosis individuals: a scoping review. J Telemed Telecare. 2020;26(1-2):14-20. doi:10.1177/1357633X18794100 30134781

[24] Schulze LN, Stentzel U, Leipert J, . Improving medication adherence with telemedicine for adults with severe mental illness. Psychiatr Serv. 2019;70(3):225-228. doi:10.1176/appi.ps.201800286 30651059

[25] Wang B, Huskamp HA, Rose S, . Association between telemedicine use in nonmetropolitan counties and quality of care received by Medicare beneficiaries with serious mental illness. JAMA Netw Open. 2022;5(6):e2218730. doi:10.1001/jamanetworkopen.2022.18730 35759264 PMC9237790

[26] Zhang T, Mosier J, Subbian V. Identifying barriers to and opportunities for telehealth implementation amidst the COVID-19 pandemic by using a human factors approach: a leap into the future of health care delivery? JMIR Hum Factors. 2021;8(2):e24860. doi:10.2196/24860 33779566 PMC8041052

[27] Lieneck C, Weaver E, Maryon T. Outpatient telehealth implementation in the United States during the COVID-19 global pandemic: a systematic review. Medicina (Kaunas). 2021;57(5):462. doi:10.3390/medicina57050462 34065050 PMC8151030

[28] Chronic Conditions Data Warehouse. Chronic conditions. HealthAPT. Accessed December 11, 2022. https://www2.ccwdata.org/documents/10280/19139421/ccw-chronic-condition-algorithms.pdf

[29] Filice CE, Joynt KE. Examining race and ethnicity information in Medicare administrative data. Med Care. 2017;55(12):e170-e176. doi:10.1097/MLR.0000000000000608 29135782

[30] Richard JV, Huskamp HA, Barnett ML, Busch AB, Mehrotra A. A methodology for identifying behavioral health advanced practice registered nurses in administrative claims. Health Serv Res. 2022;57(4):973-978. doi:10.1111/1475-6773.13974 35332555 PMC9264473

[31] Baker LC, Bundorf MK, Royalty A. Measuring physician practice competition using Medicare data. In: Aizcorbe A, Baker C, Berndt ER, Cutler DM, eds. Measuring and Modeling Health Care Costs. University of Chicago Press; 2016:351-377.

[32] Cohn ER, Zubizarreta JR. Profile matching for the generalization and personalization of causal inferences. Epidemiology. 2022;33(5):678-688. doi:10.1097/EDE.0000000000001517 35766404

[33] Hailu R, Uscher-Pines L, Ganguli I, Huskamp HA, Mehrotra A. Audio-only telemedicine visits: flaws in the underlying data make it hard to assess their use and impact. Health Affairs Forefront; July 15, 2022. Accessed December 13, 2022. https://www.healthaffairs.org/content/forefront/audio-only-telemedicine-visits-flaws-underlying-data-make-hard-assess-their-use-and

[34] Kirby JB, Zuvekas SH, Borsky AE, Ngo-Metzger Q. Rural residents with mental health needs have fewer care visits than urban counterparts. Health Aff (Millwood). 2019;38(12):2057-2060. doi:10.1377/hlthaff.2019.00369 31794321

[35] Bishop TF, Press MJ, Keyhani S, Pincus HA. Acceptance of insurance by psychiatrists and the implications for access to mental health care. JAMA Psychiatry. 2014;71(2):176-181. doi:10.1001/jamapsychiatry.2013.2862 24337499 PMC3967759

[36] Cole MB, Lee EK, Davoust M, Carey K, Kim JH. Comparison of visit rates before vs after telehealth expansion among patients with mental health diagnoses treated at federally qualified health centers. JAMA Netw Open. 2022;5(11):e2242059. doi:10.1001/jamanetworkopen.2022.42059 36378314 PMC9667322

[37] Shah VV, Villaflores CW, Chuong LH, . Association Between in-person vs telehealth follow-up and rates of repeated hospital visits among patients seen in the emergency department. JAMA Netw Open. 2022;5(10):e2237783. doi:10.1001/jamanetworkopen.2022.37783 36282505 PMC9597390

[38] Li KY, Ng S, Zhu Z, McCullough JS, Kocher KE, Ellimoottil C. Association between primary care practice telehealth use and acute care visits for ambulatory care–sensitive conditions during COVID-19. JAMA Netw Open. 2022;5(3):e225484. doi:10.1001/jamanetworkopen.2022.5484 35357448 PMC8972029

[39] Hatef E, Lans D, Bandeian S, Lasser EC, Goldsack J, Weiner JP. Outcomes of in-person and telehealth ambulatory encounters during COVID-19 within a large commercially insured cohort. JAMA Netw Open. 2022;5(4):e228954. doi:10.1001/jamanetworkopen.2022.8954 35471570 PMC9044109

[40] Wicklund E. CMS expands coverage for telehealth in mental health care. Health Leaders. Published November 5, 2021. Accessed December 11, 2022. https://www.healthleadersmedia.com/technology/cms-expands-coverage-telehealth-mental-health-care

[41] Jercich K. Massachusetts governor signs law safeguarding telehealth coverage. Healthcare IT News. Published January 4, 2021. Accessed December 11, 2022. https://www.healthcareitnews.com/news/massachusetts-governor-signs-law-safeguarding-telehealth-coverage

[42] US Department of Health and Human Services Office of Inspector General Data Brief. Inaccuracies in Medicare’s race and ethnicity data hinder the ability to assess health disparities. Published June 2022. Accessed September 25, 2023. https://oig.hhs.gov/oei/reports/OEI-02-21-00100.pdf

