# Supplementary material for: Use of Telemedicine and Quality of Care Among Medicare Enrollees With Serious Mental Illness
Source: JAMA Health Forum. 2023 Oct 27;4(10):e233648. doi: 10.1001/jamahealthforum.2023.3648 (PMC10611994; doi:10.1001/jamahealthforum.2023.3648)
Supplement: Supplement 1. — eMethods 1. Details on Study Design eMethods 2. Patient Characteristics and Outcome Definitions eMethods 3. Details on Codes Used to Identify Visits eMethods 4. Details on Model Specifications eMethods 5. Year 1 Trends and Sample Composition Shifts eResults. Details on Specialty Mental Health Visits eFigure 1. Flowchart of Cohort Exclusions and Sample Sizes eFigure 2. Share of Total Mental Health Visits Delivered With Telemedicine Over the First Year of the Pandemic eTable 1. Characteristics of Specialty Mental Health Practices Before Matching eTable 2. Year 1 Trends in Outcomes eTable 3. Changes in Patient Characteristics from Year 1 to Year 2 [file jamahealthforum-e233648-s001.pdf]

Supplemental Online Content

Wilcock AD, Huskamp HA, Busch AB, et al. Use of telemedicine and quality of care among Medicare enrollees with serious mental illness. *JAMA Health Forum.* 2023;4(10): e233648. doi:10.1001/jamahealthforum.2023.3648

eMethods 1. Details on Study Design

eMethods 2. Patient Characteristics and Outcome Definitions

eMethods 3. Details on Codes Used to Identify Visits

eMethods 4. Details on Model Specifications

eMethods 5. Year 1 Trends and Sample Composition Shifts

eResults. Details on Specialty Mental Health Visits

eFigure 1. Flowchart of Cohort Exclusions and Sample Sizes

eFigure 2. Share of Total Mental Health Visits Delivered With Telemedicine Over the First Year of the Pandemic

eTable 1. Characteristics of Specialty Mental Health Practices Before Matching

eTable 2. Year 1 Trends in Outcomes

eTable 3. Changes in Patient Characteristics from Year 1 to Year 2

This supplemental material has been provided by the authors to give readers additional information about their work.

eMethods 1. Details on Study Design

In this section, we provide more context and details on several components of our study design as well as an overview.

Variation in telemental health use across practices

See **eFigure 2** for the distribution. There were 14,071 practices in our sample before matching. We categorized the attributed practices based on their distribution of telemental health (TMH) use over the first year of the pandemic: “lowest-use” (0-49% telemental health use), “middle-use” (50-89%), and “highest-use” (90-100%, i.e. near-exclusive use). As shown in **eFigure 2**, the 3 categories were roughly similar in numbers of practices (lowest=4,747, middle=4,279, highest=5,045) and allowed us to measure changes in outcomes before and after the pandemic for patients in majority TMH use (middle) or near exclusive TMH use (highest) practices compared with patients in largely in-person care (i.e., lowest) practices.

Share of all visits provided by the attributed specialty mental health practice

As described in the Methods section of the paper, we attributed SMI patients to the specialty mental health practice that delivered the majority of their specialty mental health visits in 2019. Most SMI patients (77%) were cared for by only 1 practice (**eMethods Figure A**); only 4% of patients did not have a majority practice.

eMethods Figure A: Distribution of largest practice shares for SMI patients

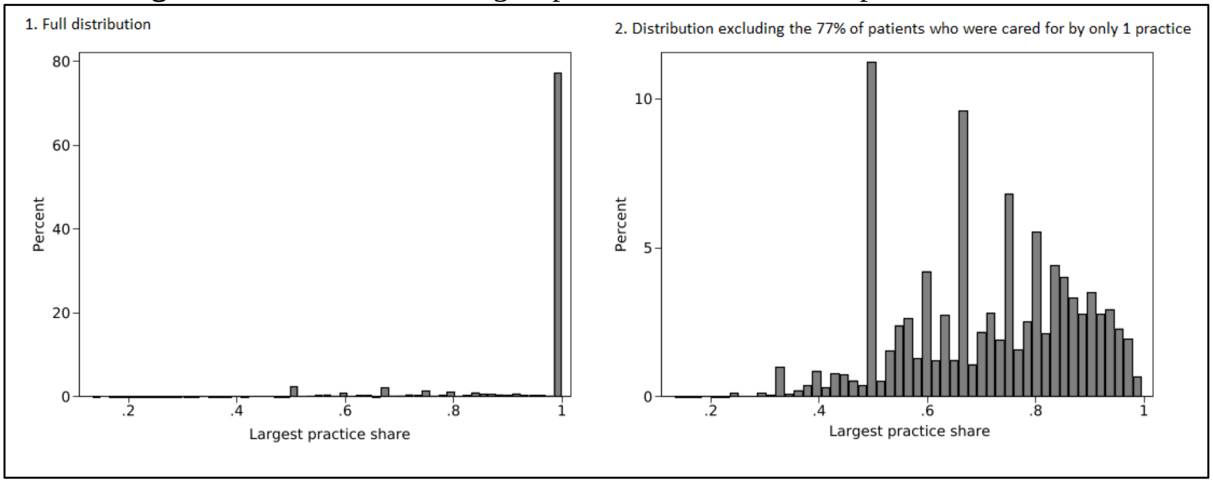

Why we used both a pre-pandemic cohort and a pandemic cohort

Following the pandemic cohort over Years 1 and 2 allowed us to measure differential changes in outcomes between highest and lowest (or middle and lowest) TMH practices. We could have only focused on the pandemic cohort to make our comparisons, however, doing so would limit our interpretation our estimates.

A concern with a pandemic cohort only design is that the use of care in the second year would be lower than the first year (when they are identified), because they were identified when they sought care. This is a common phenomenon broadly known as regression to the mean. It is unclear if changes in outcomes we observe for the control group (lowest TMH use) after the pandemic started were due to regression to the mean or due to the changed environment of the pandemic. We depict this scenario in **eMethods Figure B** (panel 1), where the treated group's visits stay the same in the year after the pandemic, while the control group's visits fall. If we assume (panel 2.i) no changes in the outcome without treatment in normal, non-study years, then we might interpret the effect of treatment (i.e., more telemedicine) was to prevent fewer visits due to broader effects of the pandemic such as social distancing. However, if we assume there's normally regression to the mean between Year 1 and Year 2 (panel 2.ii), then we would interpret the effect of treatment to be more visits than usual.

eMethods Figure B: Hypothetical changes in visits during the pandemic compared to non-study years with and without regression to the mean

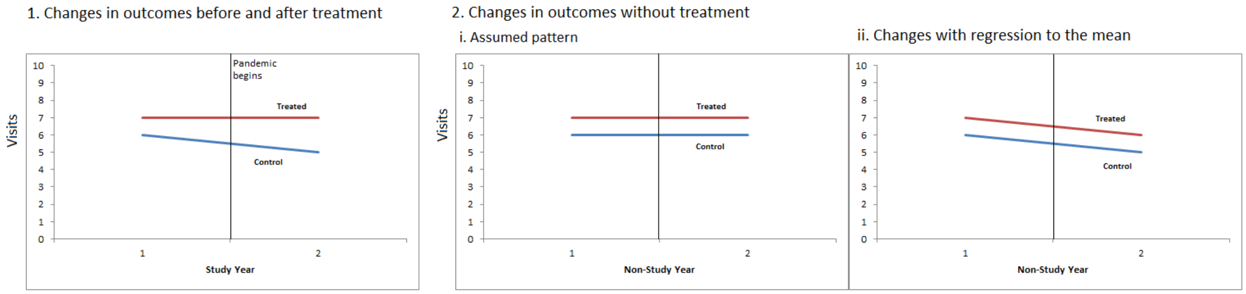

Overview of study design

While our approach adding a cohort from prior years has been used in a body of other research in other disciplines,[1] we recognize it is less common in the medical literature. We provide this overview to help clarify the design for readers.

We employed a longitudinal cohort design, where SMI patients were identified and attributed to specialty mental health practices 2019, before the pandemic started, then followed for 2 years from March 2019 through February 2020 (Year 1), and March 2020 through February 2021 (Year 2). We refer to this group of patients as the "*pandemic cohort*", which were SMI patients that experienced the first year of the pandemic and the shift towards greater TMH use.

Using the same criteria we used for the pandemic cohort, SMI patients were identified and attributed to specialty mental health practices in 2018 then followed for 2 years from March 2018 through February 2019 (Year 1), and March 2019 through February 2020 (Year 2). We refer to this group of patients as the "*pre-pandemic cohort*," which was SMI patients that did not experience the pandemic or the shift towards greater TMH use. As described in the Methods section, only practices that had at least 1 attributed SMI patient from both pandemic and pre-pandemic cohorts were included in our final analytic sample to ensure the same set of practices were present in both sets of evaluation years.

[1] Saretsky, G. (1972). The OEO P.C. Experiment and the John Henry Effect. *The Phi Delta Kappan*, *53*(9), 579–581.

eMethods Figure C depicts our approach below in 2 diagrams. In panel 1, we present a timeline for the pandemic and pre-pandemic cohorts' Year 1 and Year 2 evaluation windows. The pre-pandemic cohort study period starts in 2018 and ends before the public health emergency is declared in March 2020. The pandemic cohort starts in 2019 and ends 1 year after the pandemic started in February 2021.

eMethods Figure C: Overview of study design

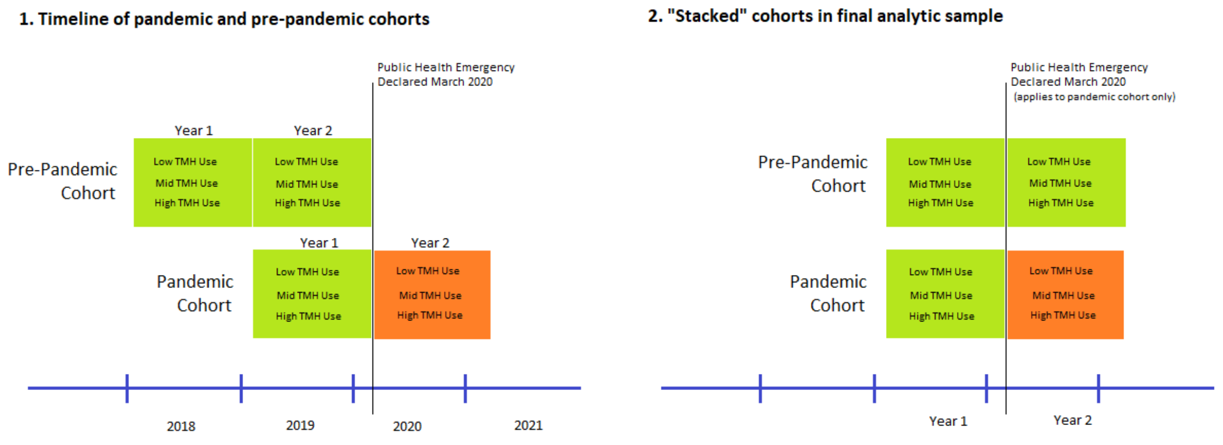

When we create our final analytic sample (panel 2), we "stack" the cohorts so that they seemingly occur contemporaneously, each with a pre-period Year 1 and a post-period Year 2.

eMethods 2. Patient Characteristic and Outcome Definitions

Patient Characteristics

We extracted demographic and prior disease burden information from the Master Beneficiary Summary Files. Characteristics were defined in the baseline identification year (2019 for the pandemic cohort, 2018 for the pre-pandemic cohort). Characteristics included age (taken at the end of the year), documented sex (male, female), race/ethnicity (non-Hispanic white or other including Asian/Pacific Islander, Black, Hispanic, American Indian/Alaska Native, and Unknown), urban versus rural residence (set by the metropolitan versus non-metropolitan status of the Rural-Urban Continuum Code for the patient's zip code), original Medicare eligibility category (age, disability, or end stage renal disease), and whether they were concurrently dually-eligible for Medicaid or not during any month of the year. From the Chronic Conditions segment, we counted up the number of chronic conditions (out of 27 chronic conditions they track[2]) for each patient. Conditions had to be established before the baseline year (before 2019 for the pandemic cohort; before 2018 for the pre-pandemic cohort).

Outcome Definitions

We evaluated outcomes that captured changes in utilization and care quality. Our primary measure of utilization was mental health visits. Our quality outcomes are approximations of well-established measures of quality tailored to fit our study design and data. The following table provides details on each study outcome and justifications for the choice of the quality measures.

<table>
<tr><th>Measure</th><th>Notes</th></tr>
<tr><td>Mental health visits with a mental health specialty clinician (in-person or telemedicine)</td><td rowspan="3">We focused on visits to mental health care specialty clinicians, because schizophrenia and bipolar I disorder typically need specialty management.

We measured what fraction of the cohort had at least 1 mental health specialty visit in the first 6 months and the second 6 months as a minimum threshold of engagement; the Veterans Affairs national health system has added a similarly structured performance measure to its national evaluation systems used for mental health care quality management.[3]

Because of concerns that 2 visits could be too low a threshold for sufficient use for patients with schizophrenia and bipolar I disorder, we</td></tr>
<tr><td>Total mental health visits with any clinician</td></tr>
<tr><td>Percentage of cohort with at least 1 mental health specialty visit in first 6 months of year and 1 visit in second 6 months of year</td></tr>
</table>

[2] Alzheimer's disease, Alzheimer's disease and related disorders or senile dementia, anemia, asthma, atrial fibrillation, benign prostatic hyperplasia, breast cancer, cataract, chronic kidney disease, chronic obstructive pulmonary disease, colorectal cancer, depression, diabetes, endometrial cancer, glaucoma, heart failure, hip or pelvic fracture, hyperlipidemia, hypertension, hypothyroidism, ischemic heart disease, lung cancer, osteoporosis, prostate cancer, acute myocardial infarction, rheumatoid arthritis, and stroke or transient ischemic attack.

[3] Lemke S, Boden MT, Kearney LK, et al. Measurement-based management of mental health quality and access in VHA: SAIL mental health domain. Psychol Serv. 2017;14(1):1-12. doi:10.1037/ser0000097; Trafton JA, Greenberg G, Harris AH, et al. VHA mental health information system: applying health information technology to monitor and facilitate implementation of VHA Uniform Mental Health Services Handbook requirements. Med Care. 2013;51(3)(suppl 1):S29-S36. doi:10.1097/MLR.0b013e31827da836

Percentage of of patients with at least 1 visit every 3 months<br>Percentage of patients with at least no mental health specialty visits in year	also measured what fraction of patients visited a mental health specialist at least once per calendar quarter.[4] Also to address the potential that during the pandemic, many patients completely dropped out of care, we measured what fraction of patients had no outpatient visits.<br><br>For each of these measures, our hypothesis was that the increased convenience of telemedicine would increase the number of visits.<br><br>We examined total mental health visits (including those from non-mental health clinicians), because we wanted to understand if there was any spillover impact of increased telemedicine visits on primary care or other mentla health visits.
Number of months with filled medications	This is a National Quality Forum–sponsored measure.[5] For patients with schizophrenia or related psychotic disorders, we focused on antipsychotic medications. For patients with bipolar-I disorder, we measured adherence to either an antipsychotic or mood-stabilizing medication. For this measure, study population further limited to those with continuous Part D coverage for the year.<br><br>Our hypothesis was that greater use of telemedicine would lead to improvements in number of visits with clinicians which, in turn, would lead to more opportunities to discuss medications and adhereance and therefore increase the number of months with medication fills.
Fraction of patients with mental health hospitalizations had an outpatient mental health service visit within 7 days of discharge	This is a National Committee for Quality Assurance measure.[6] Unit of analysis for this measure is hospitalization vs. patient-year as it is for other outcomes.<br><br>Our hypothesis is that the increased convenience of telemedicine would translate into higher rates of follow-up care.
Mental health–specific acute care use (emergency department visit or hospitalization) in a given patient year.<br>Total acute care use	Mental health acute care utilization is used by the National Committee for Quality Assurance as a quality metric.[7]<br><br>Emergency department visits and hospitalizations were classified as mental health specific if the primary diagnosis was a mental health diagnosis.<br><br>While we recognize that a hospitalization or an ED visit could be necessary for many patients, we hypothesized that, at a population level, there might be a reduction in mental health–related acute care use if patients receive more outpatient mental health care<br><br>We also examine total acute care use vs. mental illness as better management of mental illness may translate into improved outcomes

[4] Fortney J, Sullivan G, Williams K, Jackson C, Morton SC, Koegel P. Measuring continuity of care for clients of public mental health systems. Health Serv Res. 2003;38(4):1157-1175. doi:10.1111/1475-6773.00168

[5] National Quality Forum. 2021. Accessed March 25, 2022. https://www.qualityforum.org/Qps

[6] National Committee for Quality Assurance. HEDIS measures and technical resources. Accessed March 25, 2022. https://www.ncqa.org/hedis/measures

[7] National Committee for Quality Assurance. HEDIS measures and technical resources. Accessed March 25, 2022. https://www.ncqa.org/hedis/measures/mental-health-utilization

	for other chronic illnesses.
Switching of practice	While not a validated quality measure, we wanted to capture continuity of care given its importance for this patient population. Our hypothesis was that telemedicine would decrease switching.
Mortality	Though not specifically used as a quality measure for patients with serious mental illness, the hope is that improved outpatient care could deter hospitalizations, suicidal ideation, and other complications of SMI which would translate into decreased mortality.

eMethods 3. Details on Codes Used to Identify Visits

The following listing was originally published in the Supplemental Appendix for:
Busch AB, Huskamp HA, Raja P, Rose S, Mehrotra A. Disruptions in care for Medicare beneficiaries with severe mental illness during the COVID-19 pandemic. JAMA Netw Open. 2022;5(1):e2145677. doi:10.1001/jamanetworkopen.2021.45677

**Service Category**	**CPT/HCPCS Codes**
ECT or TMS services	90867-90871
Crisis intervention services	90839, 90840, H0007, H2011, S9484, S9485, T2034
Assessments, E&M services, substance use disorders medication services	90791, 90792, 99058, 99201-99205, 99211-99215, 99241-99245, 99341-99345, 99347, 99350, 99495, 99496, G0155, G0175, G0463, G0466, G0467, G0469, G0470, H0001, H0002, H0014, H0016, H0020, H0022, H0023, H0031, H0034, H0038, H0046, H1011, H2000, H2010, H2027, H5030, M0064, T1007, T1011, T1015, T0123, T1040, T1041, T2010, T2011, Z0001
Psychotherapy services	Individual or Family: 90832-90838, 90845, 90847-90849, 90865, 90875, 90876, 90880, 90900-90902, 90904, 90906, 90908, 90910, 99510, H0004, H2019, H2020, H2032, H5010, T1006, T1012<br>Group: 90853, 90857, H0005, H5020, H5025, S9454
Intensive outpatient services	H0015, S9480
Supportive psychosocial services	97003, 97004, 99490, H0036, H0037, H0039, H0040, H2001, H2013-H2018, H2021-H2026, H2030, H2031, H5220, H5230, H5240, H5299, S9127, T1017, T2012-T2015, T2018-T2023, Z0002
Screening and preventative counseling or services	98960-98962, 99078, 99385-99387, 99395-99397, 99401-99404, 99408, 99409, 99411, 99412, 99420, G0396, G0397, G0442, G0443, G0513-G0515, H0028, H0029, H0049, H0050
Codes specific for telemedicine visits	Audio only: 99441-99443, 98966-98968<br>Video: G2025 or outpatient visit with modifier code (modifiers GQ, GT or 95)
Emergency Department Codes	90500, 90510, 90515, 90517, 90520, 90530, 90540, 90550, 90560, 90570, 90580, 99281-99285

eMethods 4. Details on Model Specifications

Difference-in-Difference Specification
All difference-in-differences models used in this paper were estimated using linear regression and employed clustered standard errors at the practice level.

Model specification was the following

$$\Delta Outcome_{ip} = \beta_0 + \beta_1 Cohort_p + \beta_2 Treatment_i + \beta_3 Cohort_p * Treatment_i + X_i\tau + \varepsilon_{ip}$$

- $\Delta Outcome_{ip}$ is the change in outcome between Year 1 and Year 2 for patient i of cohort p ($\Delta Outcome_{ip} = Outcome_{ip,Year2} - Outcome_{ip,Year1}$)
    - For the pandemic cohort, Year 1 went from March 2019 through February 2020 and Year 2 went from March 2020 through February 2021
    - For the pre-pandemic cohort Year 1 went from March 2018 through February 2019 and Year 2 went from March 2019 through February 2020
- β_0 is a constant
- $Cohort_p$ is an indicator equal to 1 for the pandemic cohort and 0 for the pre-pandemic cohort
- $Treatment_i$ are dichotomous indicators for whether patient i was attributed to a lowest, middle or highest TMH use practice (the indicator for low is omitted from the model), equal to 1 if they were attributed and 0 otherwise
- $Cohort_p * Treatment_i$ are the interactions between belonging to the pandemic cohort and each treatment indicator, including middle and highest TMH use
    - The coefficients on each interaction measure are the estimates of the differential changes reported in Table 2
- ε_{ip} is the error with practice-level clustering
- X_i are beneficiary demographics (defined in 2019 for the pandemic cohort, and 2018 for the pre-pandemic cohort):
    - Bipolar indicator (1 for whether or not patient i was identified with bipolar I, 0 for schizophrenia)
    - Age indicators (<40, 40 to 54, 55 to 64, 65 to 74, and 75+)
    - Female indicator
    - Non-white indicator (Asian/Pacific Islander, Black, Hispanic, American Indian/Alaska Native, and Unknown are non-white)
    - Medicaid eligibility status (dual eligible or not) in any month
    - Original entitlement reason indicators (age 65+, disability or end-stage renal disease)
    - Metro residence indicator set equal to 1 if the bene's residence Zip code is located within Rural-Urban Commuting Areas (RUCAs) 1-3 (i.e., metropolitan area), and 0 otherwise
    - Count of 27 chronic condition indicators (0 to 1, 2 to 6, 7 to 9, and 10+)

- Conditions were counted if they were identified prior to the start of the pre-period (January 2019 for the pandemic cohort; January 2018 for the pre-pandemic cohort) and each was coded as 1 if patient i had it and 0 otherwise
- Conditions included: Alzheimer's disease, Alzheimer's disease and related disorders or senile dementia, anemia, asthma, atrial fibrillation, benign prostatic hyperplasia, breast cancer, cataract, chronic kidney disease, chronic obstructive pulmonary disease, colorectal cancer, depression, diabetes, endometrial cancer, glaucoma, heart failure, hip or pelvic fracture, hyperlipidemia, hypertension, hypothyroidism, ischemic heart disease, lung cancer, osteoporosis, prostate cancer, acute myocardial infarction, rheumatoid arthritis, and stroke or transient ischemic attack.

Differences in treatment effects by patient characteristics

We explored heterogeneity of the differential changes in visits across key patient groups including type of mental illness (schizophrenia, bipolar-I), age, race, sex, rural/urban, dual enrollment in Medicaid (marker of low income), and comorbidy counts. These estimates were presented in Figure 3 of the paper. To create these estimates we used same framework described above for our main model except each characteristic we evaluated was run in its own model and was interacted with our $Cohort_p$, $Treatment_i$, and $Cohort_p * Treatment_i$ variables. The remaining patient characteristics in X_i were kept in the model without interaction.

$$\Delta Visits_{ip} = \beta_0 + \beta_1 Cohort_p + \beta_2 Treatment_i + \beta_3 Cohort_p * Treatment_i + \beta_4 Demo_i * Cohort_p + \beta_5 Demo_i * Treatment_i + \beta_6 Demo_i * Cohort_p * Treatment_i + X_i \tau + \varepsilon_{ip}$$

To create the relative changes we report in **Figure 3**, we did the following:

1. Predict linear combinations from the equation above:
$$\beta_3 Cohort_p * Treatment_i + \beta_6 Demo_i * Cohort_p * Treatment_i$$
2. Divide the predictions from (1) by the average of the lowest group in Year 1 of the pandemic cohort for each demographic and report as percentage.

eMethods 5. Year 1 Trends and Sample Composition Shifts

We used difference-in-differences to measure the differential changes in our outcomes. In this section, we examine several methodological issues that impact the choice of this analytic strategy:

- Pre-period differential trends in outcomes
- Post-period compositional shifts in patients due to mortality

Pre-Period Trends

Difference-in-differences designs make the assumption that post-period differential changes in outcomes are the result of treatment and are not a continuation of pre-existing trends. If they were, then this would introduce a competing hypothesis for why outcomes changed that is unrelated to the intervention. And while it's impossible to prove that pre-period differential trends exist, we can test for whether a pre-period trend did not exist (null-hypothesis = 0 differential trend in the pre-period). Rejecting the null-hypothesis would suggest a differential trend existed prior to the intervention. Demonstrating no pre-period trends, therefore, is an important condition to show when using difference-in-differences analyses.

We estimated differences in monthly trends between TMH groups (middle vs lowest, and highest vs lowest) for each of our outcomes over the pandemic cohort's 12 pre-period months. Some outcomes (mortality, zero-visits and practice switching) were only available in the post-periods (**eMethods 2**) and were not evaluated. For 3 and 6 month minimums of 1 visit, we first collapsed the months into 3 and 6 month units per patient to better fit the increments of each outcome. All models used linear regression and employed clustered standard errors at the practice level.

Model specification was the following

$$Outcome_{im} = \beta_0 + \beta_1 Month_m + \beta_2 Treatment_i + \beta_3 Month_m * Treatment_i + X_i\tau + \varepsilon_{im}$$

- $Outcome_{im}$ is patient i's monthly outcome value
- β_0 is a constant
- $Months_m$ are the count of months from the start of the pre-period
    - For the pandemic cohort this is March 2019 through February 2020, or 1 through 12 months
- $Treatment_i$ are dichotomous indicators for whether or not patient i was attributed to a lowest, middle or highest TMH use practice (the indicator for lowest is omitted from the model), equal to 1 if they were attributed and 0 otherwise
- $Month_m * Treatment_i$ are the interactions between count of months and each treatment indicator, including mid and high TMH use
    - The coefficients on each interaction measure are the estimates of differential pre-period trends
- ε_{im} is the error with practice-level clustering
- X_i are beneficiary demographics (defined in 2019 for the pandemic cohort):

- Bipolar indicator (1 for whether or not patient i was identified with bipolar I, 0 for schizophrenia)
- Age indicators (<40, 40 to 54, 55 to 64, 65 to 74, and 75+)
- Female indicator
- Non-white indicator (Asian/Pacific Islander, Black, Hispanic, American Indian/Alaska Native, and Unknown are non-white)
- Medicaid eligibility status (dual eligible or not) in any month
- Original entitlement reason indicators (age 65+, disability or end-stage renal disease)
- Metro residence indicator set equal to 1 if the bene's residence Zip code is located within Rural-Urban Commuting Areas (RUCAs) 1-3 (i.e., metropolitan area), and 0 otherwise
- Count of 27 chronic condition indicators (0 to 1, 2 to 6, 7 to 9, and 10+)
    - Conditions were counted if they were identified prior to the start of the pre-period (January 2019 for the pandemic cohort) and each was coded as 1 if patient i had it and 0 otherwise
    - Conditions included: Alzheimer's disease, Alzheimer's disease and related disorders or senile dementia, anemia, asthma, atrial fibrillation, benign prostatic hyperplasia, breast cancer, cataract, chronic kidney disease, chronic obstructive pulmonary disease, colorectal cancer, depression, diabetes, endometrial cancer, glaucoma, heart failure, hip or pelvic fracture, hyperlipidemia, hypertension, hypothyroidism, ischemic heart disease, lung cancer, osteoporosis, prostate cancer, acute myocardial infarction, rheumatoid arthritis, and stroke or transient ischemic attack.

eTable 2 below presents the estimates and p values for our pre-period trend models. We found no differences in trends for any of our outcomes between middle vs. lowest, or highest vs. lowest practices.

Sample Composition Shifts due to Mortality
Another assumption in difference-in-differences is that the composition of the treated and control groups does not differentially change in the post-period. If it does, then the differential changes in outcomes may reflect (partially or entirety) the differential changes in the sample. In our cohort design, by definition all SMI patients are included in Year 1 but it would be possible that excess mortality in Year 2 could change the composition of the sample differentially between TMH groups. We did not find evidence of mortality differences (Table 2 in the main paper), but we still wanted to check to see if individual characteristics of our practice cohorts may have changed differentially.

To check for sample composition shifts, we constructed a panel dataset for our pandemic cohort that included an observation for each patient in Year 1, then a second observation for each patient that lived through Year 2. If the patient died in Year 2, then they would only have a Year 1 observation and would not contribute to the sample composition at the end of Year 2. Taking each patient characteristic one at a time, we used linear regression with errors clustered at the practice level and the following model specification

$$Characteristic_i = \beta_0 + \beta_1 Year_t + \beta_2 Treatment_i + \beta_3 Year_t * Treatment_i + \varepsilon_{it}$$

- $Characteristic_i$ is patient i's characteristic value in Year 1
  - Characteristics were included as dichotomous outcomes equal to 1 if the patient has the characteristic and 0 otherwise
  - We evaluated changes for all of out Table 1 characteristics, including:
- β_0 is a constant
- $Year_t$ is an indicator equal to 1 for Year 2 and 0 for Year 1
- $Treatment_i$ are dichotomous indicators for whether or not patient i was attributed to a lowest, middle or highest TMH use practice (the indicator for lowest is omitted from the model), equal to 1 if they were attributed and 0 otherwise
- $Year_t * Treatment_i$ are the interactions between Year 2 and each treatment indicator, including mid and high TMH use
  - The coefficients on each interaction measure are the estimates of differential changes in pre-period trends
- ε_{it} is the error with practice-level clustering

eTable 3 below presents our estimates and p values for the sample composition changes model. There were no differential changes across the array of patient characteristics we examined. The exception is that there were differentially more 75+ year olds in the highest telemedicine use practices compared to the lowest. This difference was relatively small and overall age was no different between the patients under the care of the highest and lowest practices (0.11 years; p=0.12). Nonetheless, this differential change in sample composition should be considered when interpreting our findings though we should note that we do include age as a covariate in our regression models.

eResults. Details on Specialty Mental Health Visits

We found that mental health visits differentially increased for middle and highest TMH use practices, driven almost entirely by changes in specialty mental health visits (**Table 2**). To better understand which visits changed, we identified and split specialty mental health visits into those delivered by psychiatrists, neuropsychiatrists or mental health nurse practitioners (i.e., visits where medications may have been prescribed), and those visits delivered by psychologists, clinical psychologists or licensed clinical social workers (i.e., counseling and therapy visits only). As shown in **eResults Table A**, SMI patients had more non-prescriber visits at baseline (8.5 vs 5.6 visits). Differential changes in visits for the highest TMH practices were larger from non-prescribers, corresponding to a 15.8% (95% CI 8.9, 22.7) relative increase over baseline; visits from prescribers went up 9.5% (95% CI 4.5, 14.5). For middle TMH practices, non-prescriber visits went up 8.2% (95% CI 1.4, 15.0) while prescriber visits were no different (4.6% (95% CI -0.4, 9.5)).

eResults Table A: Adjusted differential changes in specialty mental health visits by type of provider, those that can prescribe medications and those that can only deliver counseling and therapy visits

**Specialty Mental Health Visits**	**Pandemic Cohort Lowest TMH use** Year 1 Mean	**Middle v Lowest** Differential (95% CI)	**Highest v Lowest** Differential (95% CI)
with prescribers including psychiatrists, neuropsychiatrists, or nurse practitioners	5.59	0.26 (-0.02, 0.53)	0.53 (0.25, 0.81)
without prescribers including psychologists, clinical psychologists, or licensed clinical social workers	8.45	0.69 (0.12, 1.27)	1.33 (0.76, 1.91)

eFigure 1: Flowchart of Cohort Exclusions and Sample Sizes

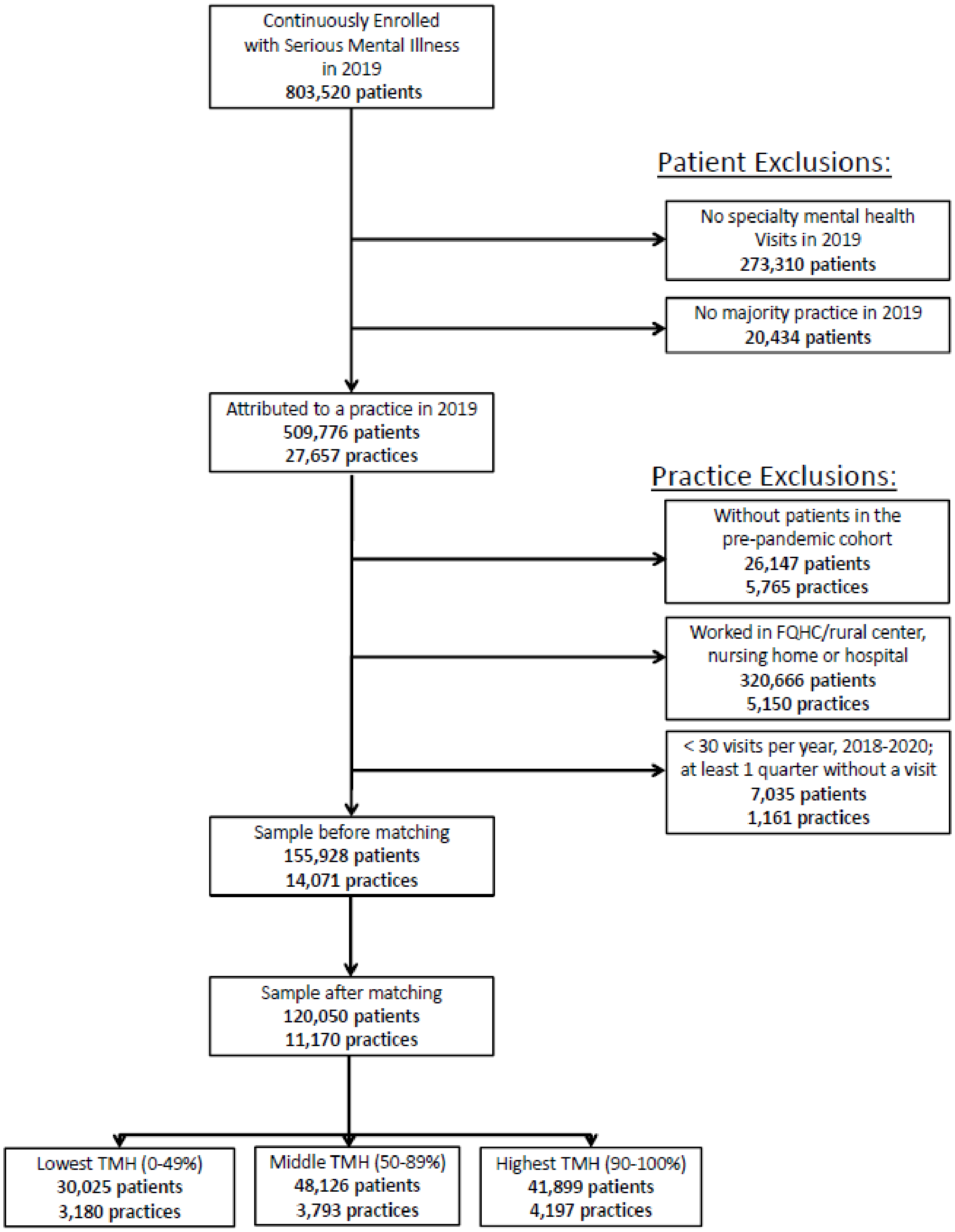

eFigure 2: Share of Total Mental Health Visits Delivered With Telemedicine Over the First Year of the Pandemic

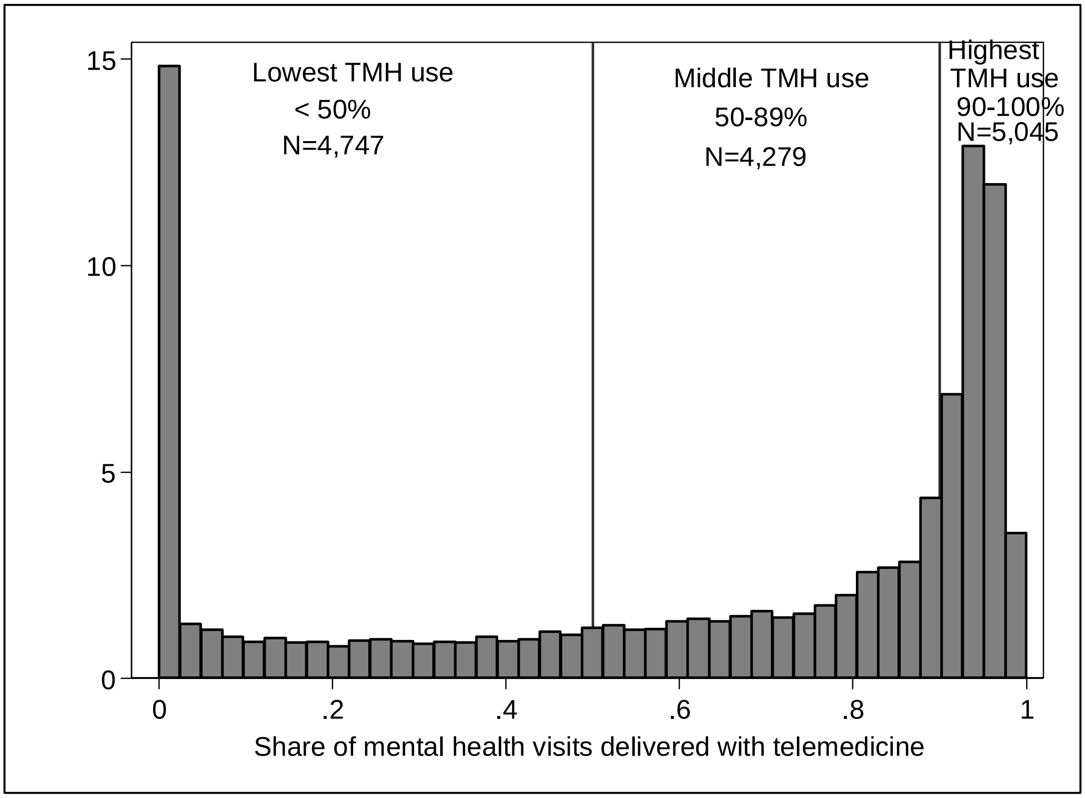

eTable 1: Characteristics of Specialty Mental Health Practices Before Matching

	Telemental Health Use March 2020 through February 2021			Absolute Standardized Differences in Means	
	Low 0 to 49%	Mid 50 to 89%	High 90 to 100%	Low vs. Mid	Low vs. High
**2019 Practice Characteristics**					
Practices, no.	4,747	4,279	5,045		
Ave. Mental Health Visits per practice, no.	450	640	519	0.188	0.072
Ave. Telemedicine Share 1st year of pandemic	14.6%	74.0%	94.7%	n/a	n/a
Practice Location					
Northeast	23.7%	29.5%	43.3%	0.132	0.425
Midwest	20.5%	22.6%	15.6%	0.052	0.127
South	34.6%	30.9%	23.9%	0.078	0.237
West	21.3%	17.0%	17.2%	0.109	0.103
Practice Size					
Solo clinician	79.1%	64.5%	79.8%	0.328	0.019
2-9 clinicians	18.1%	26.8%	15.4%	0.208	0.073
10+ clinicians	2.8%	8.7%	4.8%	0.257	0.103
Mental health focus	95.4%	95.3%	97.0%	0.005	0.085
Practice Urbanicity	85.9%	87.7%	91.8%	0.054	0.189
Medicaid focus	31.9%	32.3%	26.1%	0.013	0.212

eTable 2: Year 1 Trends in Outcomes

	Middle vs. Lowest		Highest vs. Lowest	
	Estimate	P Value	Estimate	P Value
Total mental health visits	0.003	0.157	0.002	0.387
by MH specialists	0.001	0.532	0.000	0.796
Total hospital encounters	-0.00009	0.769	-0.00008	0.356
for mental illness	-0.00006	0.584	-0.0002	0.621
Had antipsychotic or mood stabilizing medication fill in month	0.01%	0.718	-0.03%	0.336
Minimum threshold of MH visits, > 0 visits				
At least 1 visit every 3 months	-0.05%	0.826	-0.21%	0.301
At least 1 visit every 6 months	0.00%	0.990	0.11%	0.735
Follow-up after discharge				
Within 7 days	0.34%	0.243	0.04%	0.900
Within 30 days	-0.03%	0.903	-0.28%	0.317

eTable 3: Changes in Patient Characteristics from Year 1 to Year 2

	Middle vs. Lowest		Highest vs. Lowest	
	Estimate	P Value	Estimate	P Value
Bipolar-I	-0.18%	0.435	-0.15%	0.520
Age	0.06	0.399	0.11	0.122
Under 40	-0.01%	0.928	-0.10%	0.518
40-54	-0.06%	0.777	-0.08%	0.690
55-64	-0.07%	0.682	-0.12%	0.489
65-74	-0.08%	0.629	-0.01%	0.942
75+	0.23%	0.125	0.32%	0.035
Race/Ethnicity				
White	-0.03%	0.886	0.02%	0.924
Black	0.05%	0.744	0.02%	0.894
Asian/Pacific Islander	0.01%	0.882	0.01%	0.822
Other race+	-0.03%	0.679	-0.04%	0.606
Hispanic	0.00%	0.966	-0.01%	0.857
Female	-0.09%	0.682	-0.07%	0.756
Rural residence	-0.19%	0.328	-0.21%	0.242
Dually Enrolled in Medicaid	0.01%	0.964	-0.09%	0.684
Orig. Entitlement Reason				
Disability	-0.04%	0.815	-0.18%	0.272
Total chronic conditions, no.	0.02	0.300	0.04	0.102
0-1	-0.03%	0.790	-0.07%	0.553
2-6	-0.13%	0.553	-0.22%	0.324
7-9	-0.09%	0.607	-0.04%	0.802
10+	0.25%	0.296	0.34%	0.157

+ Other race includes American Indian/Alaska Native and Unknown

© 2023 Wilcock AD, et al. *JAMA Health Forum*.
