# Supplementary material for: Use of Telemedicine and Quality of Care Among Medicare Enrollees With Serious Mental Illness
Source: JAMA Health Forum. 2023 Oct 27;4(10):e233648. doi: 10.1001/jamahealthforum.2023.3648 (PMC10611994; doi:10.1001/jamahealthforum.2023.3648)
Supplement: Supplement 2. — Data Sharing Statement [file jamahealthforum-e233648-s002.pdf]

Data Sharing Statement

Wilcock. Use of Telemedicine and Quality of Care Among Medicare Enrollees with Serious Mental Illness. *JAMA Health Forum*. Published October 27, 2023.
doi:10.1001/jamahealthforum.2023.3648

Data
Data available: No

Additional Information
Explanation for why data not available: These data are accessible with a Data Use Agreement with CMS only. Interested readers should contact RESDAC for more information on access.
